# Personality and impulsivity traits associated with problematic online gaming and poker playing

**DOI:** 10.1038/s41598-025-28965-9

**Published:** 2025-12-31

**Authors:** Eleonora Anci, Stephane Rothen, Christophe Tra, Magdalena Liberacka-Dwojak, Lucien Rochat, Monika Wiłkosc-Debczynska, Vladan Starcevic, David Berle, Christina Athanasopoulou, Germano Vera Cruz, Seung-Yup Lee, Sophia Achab, Gabriel Thorens, Daniele Zullino, Joel Billieux, Yasser Khazaal

**Affiliations:** 1 Department of Psychiatry, University of Geneva, Geneva, Switzerland; 2 Department of Psychiatry, University Hospitals of Geneva, Geneva, Switzerland; 3 Addiction Medicine, Lausanne University Hospital and Lausanne University, Lausanne, Switzerland; 4 Department of Psychiatry, University of Montreal, Montréal, QC Canada; 5 Faculty of Psychology, UniDistance Suisse, Brig, Switzerland; 6 Department of Psychology, Kazimierz Wielki University, Bydgoszcz, Poland; 7 Department of Psychiatry, ReConnecte Treatment Centre, Geneva University Hospital, Geneva, Switzerland; 8 Nepean Clinical School, Sydney Medical School, Faculty of Medicine and Health, University of Sydney, Sydney, Australia; 9 Department of Psychiatry, Nepean Hospital, Penrith, Australia; 10 School of Medicine and Psychology, Australian National University, Canberra, Australia; 11 Graduate School of Health, University of Technology Sydney, Sydney, Australia; 12 Department of Occupational Therapy, University of West Attica, Athens, Greece; 13 Department of Psychology, University of Picardie Jules Verne, Amiens, France; 14 Department of Psychiatry, College of Medicine, The Catholic University of Korea, Seoul, Republic of Korea; 15 Division of Addiction Psychiatry, Department of Psychiatry, University Hospitals of Geneva, Geneva, Switzerland; 16 Institute of Psychology, University of Lausanne, Lausanne, Switzerland; 17 Center for Excessive Gambling, Addiction Medicine, Lausanne University Hospitals (CHUV), Lausanne, Switzerland; 18 Faculty of Biology and Medicine, University of Lausanne, Lausanne, Switzerland; 19 Bugnon 23 a, 1011 Lausanne, Switzerland

**Keywords:** Gaming disorder, Gambling disorder, Impulsivity, Big five personality, Neuroscience, Psychology, Psychology

## Abstract

Online gambling and gaming are associated with Problematic Usage of the Internet (PUI) in subgroups of individuals. This study aimed to assess how different personality dimensions are associated with PUI scores of Massively Multiplayer Online Role Playing Games (MMORPG) players and online poker players, and to characterize common and specific personality traits of both groups in their association with PUI. Participants (*N* = 1144) were recruited online and assessed with the Internet Addiction Test (IAT), the Big Five Inventory and the Short UPPS-P Impulsive Behavior Scale. Data were analyzed with multiple robust linear regression models. The first model tested the associations between personality and impulsivity traits with IAT scores, while controlling for age, gender, and type of online activity. The second model included interaction terms to assess whether these associations differed between MMORPG and poker players. In model 1, neuroticism, negative urgency, positive urgency and sensation seeking were significantly and positively associated with higher IAT scores after controlling for the other personality traits, age, gender, and type of online activity. Extraversion was negatively associated with IAT scores. In Model 2, no significant difference in how these personality traits relate to IAT scores was observed between the two groups. Results highlight that traits such as neuroticism, negative urgency, positive urgency, and sensation seeking constitute potential risk factors for PUI, while extraversion might constitute a protective factor against PUI. The identified associations could be useful in understanding players’ attitudes and supporting them in gaining insight into their difficulties.

**Supplementary Information:**

The online version contains supplementary material available at 10.1038/s41598-025-28965-9.

## Introduction

In recent decades, the internet has had a massive influence on how people spend their time. It has been a central tool of contemporary societal development and connectivity, and reinvented how leisure time is defined. For example, online video games (such as Massively Multiplayer Online Role Playing Games or first-person shooter games) and online forms of gambling (e.g., online poker) have reached mainstream popularity. However, in a subgroup of vulnerable individuals, these forms of entertainment can become problematic and be considered as a manifestation of Problematic Usage of the Internet (PUI)^1^, which has been recognized as a public health issue^2,3^. PUI is an umbrella term that encompasses multiple online behaviors such as gaming, pornography, gambling, social media, etc^4,5^. While some of the earlier literature described gambling disorder, including its predominantly online forms, as a subtype of PUI^4,7^, the ICD-11 now recognizes two disorders associated with online addictive behaviors, namely Gaming Disorder, predominantly online, and Gambling Disorder, predominantly online^6^.

Although the ICD-11 distinguishes between online and offline forms of gaming and gambling, both share the same diagnostic framework, with differences mainly arising from the medium of engagement. For instance, massively multiplayer online games introduce social and reward-based dynamics that may intensify involvement in comparison to offline games. Offline gambling entails social interaction and sensory stimulation, whereas online gambling offers anonymity, accessibility, and rapid betting cycles, each shaping risk differently^8–11^.

The hallmarks of both gaming and gambling disorder are impaired control, increased priority given to the behavior, and continuation despite negative consequences^6^. Beyond clinically adjacent semiology, they share similar associated factors^4^.

To better understand the genesis and maintenance of PUI, Brand et al.^4^ formulated a theoretical model illustrating the interaction between proposed biopsychosocial factors at the core of PUI. According to the Interaction of Person-Affect-Cognition-Execution (I-PACE) model^4^, specific forms of PUI are understood as consequences of interactions between predisposing factors (e.g., genetic predispositions, personality traits, psychopathology), affective and cognitive mediators (e.g., craving, coping expectancies), and moderating variables (e.g., inhibitory control, executive functioning), which together may shift internet use from a non-problematic form of entertainment to a pervasive pattern associated with functional impairment and psychological distress.

Amongst these factors, Brand et al.^4^ suggested personality as an important predisposing characteristic leading to PIU. They also noted that specific personality traits such as impulsivity may be common to all forms of PIU, but that differential traits may also be linked to specific modalities of internet use^4^.

Although recent studies investigated players during online gambling^12,13^, to the best of our knowledge, no study so far has compared problematic involvement in online gamers versus online gamblers concerning the big-five personality model^14^ and the UPPS model of impulsivity (Urgency, lack of Premeditation, lack of Perseverance, Sensation seeking)^15^. Initially, PUI has often been studied as one entity, even though it refers to a collection of different behaviors^5,7,16^. More recent studies, however, have specifically examined the associations between personality traits and particular forms of PIU, such as gaming or gambling^17–21^. Nevertheless, direct comparative studies exploring personality correlates across these specific types of PIU remain scarce^22^. Against this background, the present study aimed to investigate the role of specific personality and impulsivity traits in two different types of PUI, namely problematic involvement in MMORPG (the game World of Warcraft [WoW]) and online poker. There may be commonality in traits that are related to a vulnerability to addiction in general^23^. However, there may also be specificity in the effect of these traits, as these two online activities under consideration display unique and different design features^24^.

This additional analysis of the available data provides an opportunity to distinguish between shared and specific personality correlates, thereby clarifying whether problematic involvement in different online activities reflects a single underlying addictive process or distinct behavior-specific vulnerabilities. Such differentiation is theoretically and clinically relevant: it may refine conceptual models of PUI by identifying transdiagnostic versus activity-specific mechanisms and guide the development of prevention and intervention strategies tailored to the motivational and structural features of each activity. In this way, the present study would contribute to a more nuanced and clinically meaningful understanding of the psychological foundations of disorders due to PUI.

It was hypothesized that: (1) some common personality traits will be associated with the severity of PUI in both groups; (2) in consideration of the important differences between game structure and reward systems, that certain personality traits will correlate differently with the severity of PUI.

## Materials and methods

### Participants

This study is based on secondary analyses of adult samples of WoW and online poker players previously recruited online^1,14^. For the online poker sample^25^, adult participants engaged in internet gambling (most frequently poker) were recruited online through advertisements posted on gambling-related websites and forums. Data were collected via an anonymous online survey following informed consent. Recruitment took place between 2012 and 2014.

For the WoW sample^e.g., 26^, adults involved in the WoW game were recruited through specialized gaming forums, guild websites, and local media in 2010. Data were collected via an anonymous online survey following informed consent.

The only exclusion criterion for both studies was a lack of consent or withdrawal of consent.

### Data collection

Questionnaires were completed anonymously online via SurveyMonkey links.

### Measures

In addition to basic socio-demographic information (age and gender), three self-reported questionnaires were considered in the present study. Note that other questionnaires, except the Big Five inventory, were included in the online survey and reported in other publications^2,27–29^.

1. Internet Addiction Test (IAT).

The Internet Addiction Test (IAT) was used to assess problematic forms of internet use and consists of 20 items^30,31^ that were originally developed to measure the severity of addictive use of the internet in general^30^. Items are rated on a 5-point Likert scale (from 1 corresponding to “rarely” to 5 denoting “always”), with higher scores indicating a greater severity of PUI. In the current study, the words “Internet” and “online” in the IAT were replaced by “game” to assess involvement in online poker and WoW (e.g., “How often do you find that you stay in-game longer than you intended? “). It has been extensively used in PUI research, notably in the field of gaming disorder^3^. A one-factor model for IAT was shown to fit the data well^32^. In the present study, the internal consistency of the scale was 0.8 for the MMORPG sample and 0.92 for the online poker sample.

2. Short - Urgency-Premeditation-Perseverance-Sensation Seeking-Positive Urgency impulsive behavior Scale (S-UPPS-P).

The S-UPPS-P is a self-report questionnaire consisting of 20 items and assessing five different impulsivity traits^33^: (1) Positive urgency (the tendency to present strong reactions while experiencing intense positive emotions; (2) Negative urgency (the tendency to present strong reactions when confronted with intense negative emotions); (3) Lack of premeditation (the tendency not to consider the consequences before acting; (4) Lack of Perseverance (the inability to stay focused on a task that may be difficult or boring and (5) Sensation seeking (the search for excitement and adventure as well as an openness to new experiences). The items were scored using a 4-point Likert scale (1 = strongly disagree to 4 = strongly agree). The factorial structure of this scale was established through confirmatory factor analysis in past research^34,35^. In the present study, the internal consistency of the subscales ranges from 0.76 to 0.87 for the MMORPG sample and range from 0.78 to 0.86 for the online poker sample.

3. The Big Five Inventory – 10 items (BFI-10).

The BFI-10^14^ consists of ten items which measure: (1) Extraversion (the tendency to behave in a gregarious and unreserved way; (2) Agreeableness (the tendency to be cooperative and friendly, giving out more positive feedback; (3) Conscientiousness (the tendency to be persistent, reliable and achievement-oriented); (4) Neuroticism (the proneness towards negative affectivity); (5) Openness to Experience (the tendency to try new things and look for new experience)^36,37^. The items were scored using a 5-point Likert scale (1 = strongly disagree to 5 = strongly agree). The factorial structure of this scale was established by confirmatory factor analysis in previous studies^36^. In the present study, the internal consistency of the subscales, was examined using the Spearman-Brown correlation since^38^the subscales are composed of two-items, ranges from 0.17 to 0.73 for the MMORPG sample and ranges from 0.12 to 0.73 for the online poker sample. Low coefficients (0.17 for Agreeableness and 0.19 for Openness to experience in the MMORPG sample and 0.12 for Openness to experience and 0.18 for Agreeableness in the online poker sample) are expected given the two-item structure of the subscales and should be interpreted with caution.

It is worth noting that the S-UPPS-P and the BFI-10 may partly overlap in some of their measured dimensions. This is due to the fact that the Big Five model of personality was the starting point to develop the UPPS-P model of impulsivity^39^. Low to moderate correlations between the UPPS-P and the Big Five dimensions were reported in some studies^40^. For instance, neuroticism is related to negative urgency^40^. Premeditation and perseverance relate to conscientiousness^41^. Finally, extraversion encompasses a broader concept than sensation seeking. However, using both models concomitantly allows a more thorough assessment of the main dimensions of personality.

### Statistical analyses

Missing data within scales were treated as follows: when less than or equal to 10% of the items of a scale were not responded to, a person-mean imputation was used, whereas participants were removed from the sample if more than 10% of items of at least one scale were not completed. Considering that no clear cut-off score on the IAT can differentiate PUI from non-problematic usage^42,43^, we used IAT scores as a continuous variable. This allowed us to capture the whole range of severity assessed by the IAT, and it is in accordance with recommendations to avoid converting scores on dimensional instruments and the corresponding continuous variables into categorical variables^44,45^.

Descriptive statistics, along with group comparisons, were performed. Chi-square tests were computed for gender, whereas the Wilcoxon test was used for continuous scales since the distributions were skewed. The effect size measured by the Glass rank biserial correlation coefficient^46^ was also provided. To model the relationship between personality traits and problematic online gaming (WoW) or gambling (online poker), multiple linear regression was used. The independent variables were age, sex, personality dimensions (BFI-10) and impulsivity traits (S-UPPS-P). The dependent variable was the total score on the IAT. A hierarchical regression strategy was chosen. In the first step, only main effects were introduced in the model. In a second step, 2-way interactions between the type of online activity and personality/impulsivity scales (S-UPPS-P and BFI-10) were entered. Since residual analysis showed outliers and violation of the normality assumption, robust linear regression was performed instead^47^. Using the variance inflation factors (VIF), no sign of multicollinearity was found. Bonferroni correction was applied.

All statistical analyses were done with R 3.6.3^48^. The Glass rank biserial correlation coefficient was computed with the *rcompanion* package (Salvatore Mangiafico (2020). rcompanion: Functions to Support Extension Education Program Evaluation. R package version 2.3.25^49^. Robust linear regression was performed using the *robustbase* package^50^.

## Results

### Sample description

A total of 1547 participants (562 online poker players and 985 WoW players) completed the questionnaires. The final sample comprised 1144 participants after participants with missing data were removed: 237 participants were online poker players (325 removed, 57.8%) and 907 participants were WoW players (78 removed, 7.9%).

### Group comparisons

The characteristics of the sample are outlined in Table 1. As depicted in Table 1, after Bonferroni correction (*p* < 0.004), the only statistically significant differences found between the two groups were those related to mean age and IAT scores. WoW players were, on average younger. Furthermore, people playing on WoW scored on average higher on IAT than people involved in online poker. They did not differ in terms of S-UPPS-P and BFI-10 scores.

**Table 1:** Sample characteristics.

	Online poker Mean (SD)/ *n* (%)	WoW Mean (SD)/ *n* (%)	*r*_g_^c^	*p*-value^d^
N	237	907		
Age	30.4 (9.7)	26 (8.0)	0.32	< 0.001 ^a^
*Gender*				0.080 ^b^
Men	218 (92.0%)	795 (87.7%)		
Women	19 (8.0%)	112 (12.3%)		
IAT	35.4 (12.2)	41.2 (13.2)	0.30	< 0.001 ^a^
*S-UPPS-P*				
Negative urgency	8.3 (2.5)	8.3 (2.8)	0.03	0.5 ^a^
Positive urgency	9.5 (2.5)	10.1 (2.6)	0.11	0.006 ^a^
Lack of premeditation	6.8 (2.1)	7.2 (2.1)	0.10	0.020 ^a^
Lack of perseverance	7.6 (2.4)	7.3 (2.4)	0.08	0.058 ^a^
Sensation seeking	10.5 (2.5)	10.7 (2.7)	0.04	0.4 ^a^
*BFI-10*				
Openness	7.1 (1.7)	7.3 (1.8)	0.06	0.2 ^a^
Conscientiousness	6.4 (1.8)	6.2 (1.8)	0.05	0.2 ^a^
Extraversion	6.1 (2.0)	5.8 (2.1)	0.09	0.032 ^a^
Agreeableness	6.6 (1.6)	6.7 (1.7)	0.06	0.2 ^a^
Neuroticism	5.3 (2.0)	5.2 (2.1)	0.03	0.5 ^a^

^a^ Wilcoxon rank sum test with continuity correlation.

^b^ Pearson’s Chi-squared test with Yates’ continuity correction.

^c^ Glass rank biserial correlation coefficient (between 0 and 1).

^d^ After Bonferroni correction, statistical significance was set at *p* < 0.004.

### Multiple robust linear regression analyses

Results of robust linear regressions are summarized in Table 2. Model 1 includes only main effects, i.e., effects of the studied variables on IAT score without consideration to the online activity type. After Bonferroni correction (*p* < 0.003), younger age was significantly associated with higher scores on IAT. Regarding personality traits, extraversion was negatively related to PUI, whereas neuroticism was significantly and positively correlated with higher IAT scores. Regarding impulsivity: negative urgency, positive urgency, and sensation seeking were significantly and positively associated with higher IAT scores after controlling for other personality traits, age, gender, and online activity type.

Model 2 includes interaction terms to assess whether personality/impulsivity traits have different effects on IAT for each of the subgroups, i.e., if online activity type influences the correlation of personality/impulsivity traits with IAT scores. After Bonferroni correction (*p* < 0.002), none of the interactions with personality dimensions had a significant effect in the model. Thus, there was no statistically significant difference in the effect of personality traits on IAT score between the two subgroups.

**Table 2:** Results of robust linear regression.

	Model 1 (without interactions)				Model 2 (with interactions)			
	Estimate	Std. Error	t value	*p*-value^a^	Estimate	Std. Error	t value	*p*-value^b^
Game (ref = online poker)	5.22	0.82	6.36	< 0.001	30.03	8.92	3.37	< 0.001
Age	– 0.10	0.04	-2.45	0.014	-0.08	0.08	-0.94	0.3
Gender (ref = male)	-1.56	1.05	-1.49	0.14	-0.13	2.80	-0.04	> 0.9
*Personality traits*								
Openness	-0.36	0.19	-1.90	0.057	0.01	0.44	0.03	> 0.9
Conscientiousness	-0.30	0.22	-1.35	0.2	0.13	0.52	0.25	0.8
Extraversion	-0.71	0.17	-4.24	< 0.001	-0.63	0.39	-1.61	0.11
Agreeableness	-0.47	0.20	-2.32	0.021	0.21	0.49	0.44	0.7
Neuroticism	0.72	0.17	4.13	< 0.001	0.94	0.41	2.28	0.023
*Impulsivity traits*								
Negative urgency	0.48	0.15	3.15	0.002	0.58	0.39	1.46	0.14
Positive urgency	0.59	0.16	3.67	< 0.001	0.02	0.38	0.04	> 0.9
Lack of premeditation	0.00	0.18	-0.01	> 0.9	0.77	0.41	1.88	0.061
Lack of perseverance	0.26	0.17	1.52	0.13	0.3	0.38	0.8	0.4
Sensation seeking	0.78	0.13	5.89	< 0.001	1.32	0.33	3.96	< 0.001
*Interactions between game (ref = online poker) and scales*								
Openness					-0.04	0.09	-0.45	0.7
Conscientiousness					-1.93	3.02	-0.64	0.5
Extraversion					-0.47	0.49	-0.97	0.3
Agreeableness					-0.55	0.58	-0.96	0.3
Neuroticism					-0.15	0.44	-0.34	0.7
Negative urgency					-0.89	0.54	-1.65	0.10
Positive urgency					-0.17	0.45	-0.38	0.7
Lack of premeditation					-0.11	0.43	-0.26	0.8
Lack of perseverance					0.67	0.42	1.61	0.11
Sensation seeking					-0.98	0.45	-2.16	0.031

^a^ After Bonferroni correction, statistical significance was set at *p* < 0.004.

^b^ After Bonferroni correction, statistical significance was set at *p* < 0.002.

## Discussion

This study aimed to assess how different personality dimensions are associated with PUI scores of people involved in playing MMORPG and online poker, and to characterize common and specific personality traits of both groups in their association with PUI. First, the MMORPG group presented with a higher IAT score than the poker group. This may be since WoW can exclusively be played online, therefore, its exclusive reliance on the internet may explain the higher IAT scores. In contrast, there are many ways to play poker that do not rely on the internet (e.g., casino, offline groups). Another possible explanation for this observation is that excessive involvement in MMORPGs has been repeatedly associated with the need for escapism.

The escapism motive for playing games has been associated with problematic involvement in video games^51,52^. According to the recently proposed *Compensatory-Dissociative Online Gaming* model (2024)^53^, gaming related PUI can develop along a continuum ranging from compensatory to dissociative forms of involvement. On this continuum, gaming may initially serve as a compensatory strategy to manage unmet psychological needs or distress through active escapism and emotional displacement, but in more maladaptive forms, it may evolve toward dissociative regulation and virtual withdrawal. Games, such as WoW are possibly, particularly conducive to such processes, as their persistent virtual worlds, customizable avatars, and immersive social environments facilitate both emotional escape and the reconstruction or extension of identity in virtual settings^54^.

Other structural features of MMORPGs and WoW in particular can contribute to addictiveness potential, such as their progression system and highly social nature (players group themselves in guilds and forums), or the fact that they take place in permanent virtual worlds.

Second, regarding the Big Five traits, we found that high neuroticism and low extraversion are associated with higher symptoms of PUI in our sample for both online poker and WoW gamers. This finding is consistent with other studies investigating the interplay of personality and PUI, while also underlining a positive correlation with psychoticism, and a negative correlation with conscientiousness and agreeableness^55^. Unlike Mak et al.^55^, we did not detect a significant correlation with conscientiousness and agreeableness. Our use of a convenience sample of individuals who willfully participated in an online survey may have impacted the representativeness of our sample. Recruited individuals may display higher conscientiousness and agreeableness than the general population of people playing poker and WoW because of such sampling bias^29^. It is not clear whether high neuroticism and low extraversion are specific factors associated to PUI or whether they act as common risk factors for addictive behaviors in general. Dash et al.^56^ conducted a study examining Big Five profiles in individuals presenting with different types of offline addictive disorders, including gambling disorder. They also found neuroticism to be a common factor for the studied type of addictions, but did not report extraversion as a protective factor. Given these results, we might wonder whether extraversion plays a specific role in online addictive behavior. A support to this hypothesis is that a large corpus of previous research showed that PUI is associated with a preference for online (versus offline) interactions^57^.

We hypothesize that the link between neuroticism and PUI may be through their association with psychopathology. Psychopathology has been associated with both problematic use of the internet^58–60^ and neuroticism^61–63^. Thus, individuals with high neuroticism levels may be more at-risk of negative affect (and psychopathology) and seek refuge in online addictive behaviors. Online video games may provide relief through escapism and offer a way of coping with negative affect^64^.

Extraversion may be of particular interest in online addictive behaviors because the internet may offer a buffer to help introverted (i.e., individuals with low extraversion) and socially anxious individuals to engage in social interactions, for example, by providing anonymity^65^. Internet use may be seen as a way of coping with loneliness, especially in socially inhibited individuals^66^. Demetrovics et al. also highlighted the social aspect of games as a major motive for the use of online games^64^. This aspect of the internet may explain why low extraversion was found to be a significant factor in our study, whereas Dash et al.’s study, which focused solely on offline addictive behaviors, did not find this association.

Third, in the S-UPPS scale, positive urgency, negative urgency, and sensation seeking are all positively correlated with higher IAT scores in both groups. This result is in accordance with findings of an association between PUI and impulsivity^58,67–69^ and between PUI and attention deficit/hyperactivity disorder^70^. It seems that impulsivity may also be a common factor for addictive disorders in general^69,71^. Rømer Thomsen et al. found UPPS-P scores, especially its urgency (an aggregate score of positive and negative urgency) and lack of perseverance components, to be positively associated with other addictive behaviors (alcohol, cannabis, other drugs, pornography, and food) in a sample of adolescents and young adults. However, the same study did not find this association with problematic videogaming^71^, which stands in contrast to our findings.

Finally, our study did not support the hypothesis that MMORPGs and online poker players differ in terms of the correlations between PUI severity and their personality/impusilivity profiles. This suggests that these personality factors may serve as a common risk factor for PUI regardless of the way in which the Internet is used (WoW or poker).

Taken together, these findings suggest that while people who play on WoW and those playing online poker may differ in the structure and motives of their online activities, the psychological mechanisms associated with PUI may share substantial commonalities. The similar personality and impulsivity profiles observed across both groups support the view that PUI reflects a set of transdiagnostic vulnerability factors—such as high neuroticism and urgency—that may underlie PUI. From a theoretical standpoint, this reinforces dimensional models of PUI emphasizing common underlying processes. Clinically, these results highlight the importance of assessing personality and emotion-regulation traits when designing prevention and intervention strategies, regardless or in addition to the specific online activity involved. Practically, interventions targeting impulsivity and maladaptive coping strategies may prove useful across diverse PUI manifestations. Future studies should explore how contextual or structural characteristics of online environments interact with these shared psychological factors to shape distinct trajectories of PUI.

### Limitations

There are several limitations of our study. Generalizability may be limited because recruitment was based on a convenience sample. The study is subject to self-selection biases, which may limit sample representativeness and increase inclusion of highly involved gamers^29^.

Moreover, information regarding participants’ country of residence was not collected, which prevents us from identifying the geographical composition of the sample. This limits our ability to examine potential cultural or regional differences in the observed associations. Self-report instruments used in the study are subject to reporting biases.

Group comparability may also be limited by the fact that we used secondary data coming from two distinct primary studies, with different recruitment methodologies. This potential limitation may explain the major differences in the proportion of incomplete data in the two groups (online poker missing data 57.8%, WoW missing data = 7.9%). In the online poker study, participants were recruited through gambling-related websites and forums. In contrast, the WoW sample was recruited through gaming forums, guild websites, and local media. These recruitment contexts may thus have influenced participants’ engagement levels, motivation for participation, and willingness to complete questionnaires. Consequently, differences in recruitment sources and respondent characteristics should be considered when interpreting between-group comparisons, as they may reflect both behavioral and contextual variations inherent to the populations studied rather than purely psychological or personality-based differences.

Our conclusions are only applicable to online poker and MMORPG players, not to other forms of PUI. Data collected on WoW players cannot be generalized to videogaming in general, because different vulnerabilities and motivations may underlie gaming genre preference and their interactions may lead to pathogenesis of gaming disorder^72^. Furthermore, it should be noted that the study did not exclude individuals who might also engage in other online games or gambling activities. Therefore, while the study focused on problematic involvement in the respective target behaviors (MMORPG playing or online poker), the possibility of overlapping gaming or gambling behaviors among participants cannot be ruled out.

A further limitation of the present study may rely on the use of the IAT, a scale developed before the ongoing progress that came from the recent ICD-11 classification related to gaming and gambling disorders. While the IAT was originally developed to assess generalized PUI, its conceptual validity has been increasingly questioned considering the evolving understanding of behavioral addictions and the specificities of online activities. For instance, ICD-11^6^ now recognize distinct disorders, such as Gaming Disorder and Gambling Disorder – predominantly online, emphasizing that PUI should be assessed within each specific online behavior. In response to these conceptual and contextual developments, the present study employed an adapted version of the IAT^73^, tailored to assess PUI in relation to a specific behavior, respectively WoW or online poker. This psychometrically assessed modified IAT^74^ aimed to enhance content validity by asking participants to give their answers based on the assessed activity.

In addition, the content of the adapted IAT was compared^75^ with the diagnostic criteria proposed in the DSM-5 for *Internet Gaming Disorder* and *Gambling Disorder*^76,77^. This comparison showed that most DSM-5 criteria were represented within the IAT, except for one criterion related to tolerance in Gaming Disorder and two criteria in Gambling Disorder (“reliance on others to provide money to relieve financial problems caused by gambling” and “chasing one’s losses”). Furthermore, several IAT items correspond to the main features highlighted in the ICD-11—namely, impaired control, increasing priority given to the behavior, and continuation despite negative consequences—although other items refer to dimensions not explicitly included in the ICD-11, such as coping motives, preoccupation, or withdrawal.

Despite this partial overlap, the IAT—even in its adapted form—does not fully capture the multidimensional structure of problematic use as conceptualized in the ICD-11. Consequently, the present findings should be interpreted with caution, within the framework of evolving diagnostic and theoretical models of PUI. Further studies would benefit from the use of new instruments^73^, like the Assessment of Criteria for Specific Internet-use Disorders relying on ICD-11 criteria^78,79^ and allowing for the assessment of different specific behaviors.

Moreover, because of the large sample size, some statistically significant results may represent small effects of limited practical importance. Besides, the relatively low internal consistency coefficients observed for some BFI-10 subscales reflect the brevity of the measure and may have attenuated the observed associations involving these traits. Finally, the study was cross-sectional by design and did not allow exploration of the causal links between variables.

### Future research

Future research should further characterize PUI subgroups, as they have been recognized as distinct entities^80,81^. Studies comparing the personality profiles of individuals engaged in internet-related behaviors with those of people involved in offline addictive behaviors (i.e. offline variants of video games; offline poker game sessions) could help further understand the specific role that the internet plays in the development of PUI, and how online addictive behaviors differ from more traditional addictions. Better understanding personality characteristics of individuals vulnerable to PUI, and what factors are shared, would be instrumental in tailoring prevention and treatment interventions that can encompass diverse types of PUI, as new internet related technologies are being created at a fast pace. We also call for future research to use PUI scales on a continuous basis, as the disorder encompasses a wide range of individuals struggling with highly variable severity of symptoms.

The present study shows that low extraversion, high neuroticism, high negative urgency, high positive urgency and high sensation seeking are correlated with the severity of two subtypes of problematic internet use (online gaming and online gambling). Conversely, high extraversion may constitute a protective factor. In these groups, common personality/impulsivity factors correlated with the severity of PUI and do not differ in both groups, suggesting that common personality factors may be more important than specific differences.

## Supplementary Information

Below is the link to the electronic supplementary material.

Supplementary Material 1

## Data Availability

Due to ethical and privacy reasons, the data used in this study are currently not made publicly available. However, access may be granted upon reasonable request, provided that appropriate data protection measures are ensured.The contact person for the data is Stephane Rothen: Stephane.rothen@psychologie.ch.
