# Supplementary material for: Personality and impulsivity traits associated with problematic online gaming and poker playing
Source: Sci Rep. 2025 Dec 31;16:129. doi: 10.1038/s41598-025-28965-9 (PMC12765006; doi:10.1038/s41598-025-28965-9)
Supplement: Supplementary file 1 — Supplementary Material 1 [file 41598_2025_28965_MOESM1_ESM.pdf]

HUG
Geneva University Hospitals
Department of Mental Health and Psychiatry
Addiction Services

<u>Questionnaire</u>

Thank you for reading and accepting the terms and conditions for participating in this survey.

Please also answer the following questions.
In these questions, when the words "gaming" « game » or « play-playing » are used, it always refers to poker (for the Poker study sample) or MMORPG gaming (for the corresponding sample).

Personal information

1. My year of birth is (e.g., 1980):
2. I am
    - A man
    - A woman

Internet Addiction Test (adapted)

1. Do you ever play longer than you intended to at the beginning?
    - Rarely
    - Occasionally
    - Frequently
    - Often
    - Always
2. Do you ever neglect your daily tasks in order to spend more time playing?
    - Rarely
    - Occasionally
    - Frequently
    - Often
    - Always
3. Do you ever prioritize the fun of the game over intimacy with your partner?
    - Rarely
    - Occasionally
    - Frequently
    - Often
    - Always
4. Do you ever form new relationships with players?
    - Rarely
    - Occasionally
    - Frequently
    - Often
    - Always
5. Do your loved ones criticize you for spending too much time playing?
    - Rarely
    - Occasionally
    - Frequently
    - Often

- Always

6. Do your grades or schoolwork ever suffer because of the time you spend playing?
    - Rarely
    - Occasionally
    - Frequently
    - Often
    - Always
7. Do you ever log into the game before completing a necessary and urgent task?
    - Rarely
    - Occasionally
    - Frequently
    - Often
    - Always
8. Does your work performance or productivity suffer because of the time you spend playing?
    - Rarely
    - Occasionally
    - Frequently
    - Often
    - Always
9. Do you ever feel defensive or refuse to answer if someone asks you what you are doing on the internet- when you are playing?
    - Rarely
    - Occasionally
    - Frequently
    - Often
    - Always
10. Do you ever find yourself chasing away the worries of everyday life with the comforting thought of going out to play ?
    - Rarely
    - Occasionally
    - Frequently
    - Often
    - Always
11. Do you ever look forward to playing again?
    - Rarely

- Occasionally
- Frequently
- Often
- Always

12. Do you ever think that life without gaming would be boring, empty, and joyless?
    - Rarely
    - Occasionally
    - Frequently
    - Often
    - Always
13. Do you ever respond in a harsh tone, yell, or get annoyed if someone disturbs you while you are playing?
    - Rarely
    - Occasionally
    - Frequently
    - Often
    - Always
14. Do you ever lose sleep because you stayed up late playing?
    - Rarely
    - Occasionally
    - Frequently
    - Often
    - Always
15. When you are not playing, do you ever actively think about or dream about playing?
    - Rarely
    - Occasionally
    - Frequently
    - Often
    - Always
16. Do you ever say to yourself, "Just a few more minutes," when you are playing?
    - Rarely
    - Occasionally
    - Frequently
    - Often

- Always

17. Do you ever try to reduce the amount of time you spend playing without succeeding?
    - Rarely
    - Occasionally
    - Frequently
    - Often
    - Always
18. Do you ever hide from others how much time you spend playing?
    - Rarely
    - Occasionally
    - Frequently
    - Often
    - Always
19. Do you ever choose to spend more time playing than spending time going out with your family and friends?
    - Rarely
    - Occasionally
    - Frequently
    - Often
    - Always
20. Do you ever feel depressed, in a bad mood, or irritable when you are not playing, and then feel better when you play?
    - Rarely
    - Occasionally
    - Frequently
    - Often
    - Always

UPPS

Below are a number of statements describing ways of behaving or thinking. For each statement, please indicate the extent to which you agree or disagree with the statement by checking the box that best applies. Make sure you indicate your agreement or disagreement for each statement below.

1. I usually think carefully before doing anything.
   - Strongly agree
   - Somewhat agree
   - Somewhat disagree
   - Strongly disagree
2. When I am really excited, I tend not to think about the consequences of my actions.
   - Strongly agree
   - Somewhat agree
   - Somewhat disagree
   - Strongly disagree
3. I sometimes like to do things that are a bit frightening.
   - Strongly agree
   - Somewhat agree
   - Somewhat disagree
   - Strongly disagree
4. When I am upset, I often act without thinking.
   - Strongly agree
   - Somewhat agree
   - Somewhat disagree
   - Strongly disagree
5. I generally like to see things through to the end.
   - Strongly agree
   - Somewhat agree
   - Somewhat disagree
   - Strongly disagree
6. My thinking is usually careful and purposeful.
   - Strongly agree
   - Somewhat agree
   - Somewhat disagree
   - Strongly disagree
7. In the heat of an argument, I will often say things that I later regret.

- Strongly agree
- Somewhat agree
- Somewhat disagree
- Strongly disagree

8. I finish what I start.
   - Strongly agree
   - Somewhat agree
   - Somewhat disagree
   - Strongly disagree
9. I quite enjoy taking risks.
   - I completely agree
   - Somewhat agree
   - Somewhat disagree
   - Strongly disagree
10. When I am in great mood, I tend to get into situations that could cause me problems.
   - Strongly agree
   - Somewhat agree
   - Somewhat disagree
   - Strongly disagree
11. I almost always finish projects that I start
   - Strongly agree
   - Somewhat agree
   - Somewhat disagree
   - Strongly disagree
12. When I am upset I often act without thinking.
   - Strongly agree
   - Somewhat agree
   - Somewhat disagree
   - Strongly disagree
13. I usually think carefully before doing anything.
   - Strongly agree
   - Somewhat agree
   - Somewhat disagree
   - Strongly disagree
14. I generally seek new and exciting experiences and sensations.
   - Strongly agree

- Somewhat agree
- Somewhat disagree
- Strongly disagree

15. I tend to act without thinking when I am really excited.
    - Strongly agree
    - Somewhat agree
    - Somewhat disagree
    - Strongly disagree
16. Unfinished tasks really bother me.
    - Strongly agree
    - Somewhat agree
    - Somewhat disagree
    - Strongly disagree
17. When I feel rejected, I will often say things that I later regret
    - Strongly agree
    - Somewhat agree
    - Somewhat disagree
    - Strongly disagree
18. I welcome new and exciting experiences and sensations, even if they are a little frightening and unconventional
    - I completely agree
    - Somewhat agree
    - Somewhat disagree
    - Strongly disagree
19. Before making up my mind, I consider all the advantages and disadvantages.
    - Strongly agree
    - Somewhat agree
    - Somewhat disagree
    - Strongly disagree
20. When I am very happy, I can't seem to stop myself from doing things that can have bad consequences.
    - Strongly agree
    - Somewhat agree
    - Somewhat disagree
    - Strongly disagree

BIFI-10

For each of the following statements, please indicate the extent to which you agree or disagree with the statement.

I see myself as someone who...

1 ... is reserved
- Disagree strongly
- Disagree a little
- Neither agree nor disagree
- Agree a little
- Agree strongly

2. ... is generally trusting
- Disagree strongly
- Disagree a little
- Neither agree nor disagree
- Agree a little
- Agree strongly

3. ... tends to be lazy
- Disagree strongly
- Disagree a little
- Neither agree nor disagree
- Agree a little
- Agree strongly

4. ... is "relaxed," handles stress well
- Disagree strongly
- Disagree a little
- Neither agree nor disagree
- Agree a little
- Agree strongly

5 ... has few artistic interests
- Disagree strongly
- Disagree a little
- Neither agree nor disagree
- Agree a little
- Agree strongly

6. ... is outgoing, sociable

- Disagree strongly
- Disagree a little
- Neither agree nor disagree
- Agree a little
- Agree strongly

7. ... tends to find fault with others
   - Disagree strongly
   - Disagree a little
   - Neither agree nor disagree
   - Agree a little
   - Agree strongly
8. ... does a thorough job
   - Disagree strongly
   - Disagree a little
   - Neither agree nor disagree
   - Agree a little
   - Agree strongly
9. ... gets nervous easily
   - Disagree strongly
   - Disagree a little
   - Neither agree nor disagree
   - Agree a little
   - Agree strongly

10 ... has an active imagination
   - Disagree strongly
   - Disagree a little
   - Neither agree nor disagree
   - Agree a little
   - Agree strongly
